# A systematic review of MRI studies examining the relationship between physical fitness and activity and the white matter of the ageing brain

**DOI:** 10.1016/j.neuroimage.2015.09.071

**Published:** 2016-05-01

**Authors:** Claire E. Sexton, Jill F. Betts, Naiara Demnitz, Helen Dawes, Klaus P. Ebmeier, Heidi Johansen-Berg

**Affiliations:** a FMRIB Centre, Nuffield Department of Clinical Neurosciences, John Radcliffe Hospital, University of Oxford, OX3 9DU, UK; b Faculty of Health and Life Sciences, Oxford Brookes University, Oxford OX3 0BP, UK; c Department of Psychiatry, Warneford Hospital, University of Oxford, OX3 7JX, UK

**Keywords:** Ageing, Fitness, Magnetic resonance imaging, Physical activity, Review, White matter

## Abstract

Higher levels of physical fitness or activity (PFA) have been shown to have beneficial effects on cognitive function and grey matter volumes in older adults. However, the relationship between PFA and the brain's white matter (WM) is not yet well established. Here, we aim to provide a comprehensive and systematic review of magnetic resonance imaging studies examining the effects of PFA on the WM of the ageing brain. Twenty-nine studies were included in the review: eleven examined WM volume, fourteen WM lesions, and nine WM microstructure. While many studies found that higher levels of PFA were associated with greater WM volumes, reduced volume or severity of WM lesions, or improved measures of WM microstructure, a number of negative findings have also been published. Meta-analyses of global measures of WM volume and WM lesion volume yielded significant, but small, effect sizes. Overall, we found evidence for cautious support of links between PFA and WM structure, and highlighted key areas for future research including the extent to which the relationship between PFA and WM structure is anatomically specific, the influence of possible confounding factors, and the relationship between PFA, WM and cognition.

## Highlights

• We review MRI studies of PFA and the WM of the ageing brain.
• Higher levels of PFA were often associated with improved WM outcomes.
• Meta-analyses yielded significant, but small, effect sizes.

## Introduction

Magnetic resonance imaging (MRI) studies have provided key insights into the macro- and micro-structures of the brain's white matter (WM) (Walhovd et al., 2014). For example, T_1_-weighted MRI studies have quantified WM volumes, with either a single global measure of WM volume assessed, or WM volume analysed on a voxel-wise basis across the whole brain; proton-density, T_2_, T_2_* or fluid attenuated inversion recovery studies have examined the volume or rating of WM lesions; and diffusion tensor imaging (DTI) studies have detailed measures of WM microstructure, including fractional anisotropy (FA), mean diffusivity (MD), axial diffusivity (AD) and radial diffusivity (RD).

Such MRI studies have played a prominent role in the characterisation of WM changes with ageing, detailing decreasing WM volumes, increasing volume and severity of WM lesions, and declining WM microstructure with advancing age (Bennett and Madden, 2014, Gunning-Dixon et al., 2009, Sexton et al., 2014). In addition to describing the relationship between age and WM measures, MRI studies have also highlighted substantial between-subject variance. As a result, there is great interest in identifying factors that can help explain such heterogeneity, as well as in interventions that could slow, prevent or even reverse, age-related decline.

Higher levels of physical fitness (PF) and physical activity (PA) have been shown to have beneficial effects on cognitive function and grey matter volumes in older adults (Bherer et al., 2013, Erickson et al., 2014). However, the relationship between physical fitness or activity (PFA) and the brain's WM is not yet well established. Here, we aim to provide a systematic report of cross-sectional and longitudinal MRI studies that have examined the effects of PFA on the WM of the ageing brain. For each aspect of WM structure (WM volume, WM lesions, WM microstructure), we summarise the results to date and perform meta-analyses of global WM measures where possible. We hypothesise that higher levels of PFA would be associated with greater WM volumes, reduced volume or severity of WM lesions, or improved measures of WM microstructure.

## Methods

### Data sources

Online searches of the databases EMBASE and MEDLINE were performed in August 2015. An example of the search strategy used in the MEDLINE database is shown in the Supplementary Material: Fig. S1. Reference lists of included studies and relevant reviews were manually searched for additional studies.

### Study selection

Two authors independently screened the title, abstracts and, where appropriate, full-text of identified citations and any disagreements were resolved by consensus. For studies to be included in the review, the following criteria had to be met:

(1) Published as a journal article or letter. While this may raise susceptibility to publication bias, restricting the search to published results serves as a guarantee of peer-reviewed quality in included reports.
(2) Assessed the level of PFA by fitness test, questionnaire or accelerometry, or administered an exercise intervention. Studies assessing mobility or motor performance were excluded, e.g. gait tests and activities of daily living questionnaires.
(3) Administered an MRI brain scan to examine one or more aspects of WM structure. Composite measures of WM spanning more than one aspect of WM structure (e.g. the single factor outcome of principal component analyses of FA and WML values in Burzynska et al., 2014) were not included, nor were DTI analyses of grey matter structure.
(4) Examined a direct association between PFA and MRI measures, a difference in MRI measures between groups that differed in PFA, or a difference in PFA measures between groups that differed in WM outcomes.
(5) Included a sample of healthy adults, with a mean age over the age of 60, who were not selected based upon the presence of medical diagnoses (including hypertension, mild cognitive impairment or dementia), or genotype. Studies that selected participants based upon PFA level, mobility or WM measures were included.

### Data extraction and analysis

Two authors independently extracted the following details using a structured data abstraction form: aspect of WM structure examined (WM volume, WM lesions, WM microstructure), anatomical specificity (global or local measure of WM structure), study design (cross-sectional or longitudinal MRI assessment, interval between PFA and MRI assessments, frequency and duration of exercise intervention), participant demographics (sample size, mean age, percentage of female participants), methods (PFA and MRI assessment) and results (statistically significant findings, p < 0.05 unless a different limit was imposed by the authors).

Where possible, results are presented after co-varying for age and sex, but before co-varying for additional factors (e.g. BMI, social class, disease), with results after additional co-varying noted in table footnotes. For studies that examined WM structure locally, regions were grouped according to cerebral lobe (frontal, temporal, parietal, occipital, limbic) or tract (corpus callosum, superior longitudinal fasciculus, etc) and any lobe or tract that contained a minimum of one region that was statistically significant is presented.

Studies with overlapping samples were only excluded when the same aspect of WM structure was examined in both papers. In such cases, preference was first given to the study with the largest sample size (Tian et al., 2014a preferred to Tian et al., 2014b). Where sample size was equal, preference was given to analyses of a global measure of WM volume expressed as a percentage of intracranial volume (ICV), rather than raw values (Tseng et al., 2013b preferred to Tseng et al., 2013a), and voxel-wise analysis was preferred to region-of-interest (ROI) analysis (Liu et al., 2012 preferred to Marks et al., 2011). When studies reported multiple MRI analysis methods in a single paper (e.g. voxel-wise analysis and tract-based spatial statistics (TBSS) (Liu et al., 2012)), results from the primary analysis method is presented in the table, with results from additional methods noted in the table footnotes. Analyses in which the directionality of the relationship between PFA and WM could not be inferred were not included (e.g. differences in the prevalence of moderate WMH score, which could reflect differences in mild or severe WMH score (Frederiksen et al., 2015)). When studies used multiple measures of PFA, results are only presented from one measure (fitness test preferred to accelerometry or questionnaire, accelerometry preferred to questionnaire, kcal/week preferred to frequency of activity). This order was chosen as a result of a recent review concluding that results from cross-sectional studies of PF on grey matter were more consistent than studies of PA (Erickson et al., 2014). Finally, when both group difference and correlational analyses were performed, the primary analyses are reported in the table, with additional results discussed in the text.

### Data synthesis

Data for global WM volume and global WM lesion volume were analysed using Comprehensive Meta-Analysis (version 2.2, © July 27 2011, Biostat Inc., Englewood, NJ). Effect size was measured using standardised mean differences (Cohen's) d, calculated from available statistical parameters, and a random-effects model was used to calculate the pooled mean effect size (Borenstein et al., 2010). Heterogeneity was assed using Cochrane's Q and publication bias was considered using Begg and Mazumdar rank correlations (Begg and Mazumdar, 1994). Finally, the influence of participants' characteristics (age, percentage female) and design (interval between PFA and MRI assessment) was analysed using fixed effect regression with Hedges' g.

As only one study analysed global WM microstructure, a meta-analysis of this measure was not performed. Local WM measures require co-ordinate-based or image-based meta-analysis methods (Salimi-Khorshidi et al., 2009), which were considered beyond the scope of this review.

## Results

### Study selection

Titles and abstracts of three hundred and ninety nine citations were screened, with twenty-nine papers included in the review. A flow diagram of the identification and attrition of studies is provided in the Supplementary Material: Fig. S2.

### Global white matter volume

A total of five studies examined the relationship between a global measure of WM volume and PFA, as summarised in Table 1.

All studies employed a cross-sectional MRI design. Although three studies did not report a significant association (Bugg and Head, 2011, Burns et al., 2008, Tseng et al., 2013b), higher levels of PA were associated with greater global WM volumes in the two largest studies (Benedict et al., 2013, Gow et al., 2012).

**Fig. 1:**
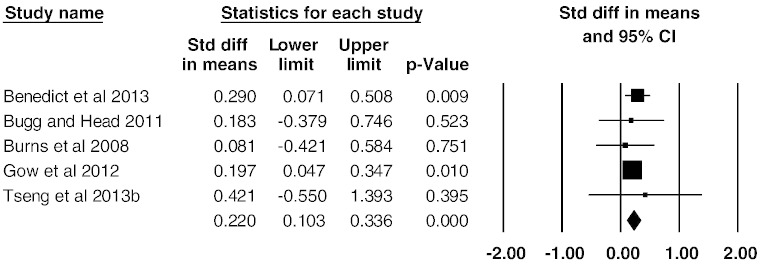
Effect sizes (Cohen's d) for global WM volume: higher PFA is associated with greater WM volumes.

**Fig. 2:**
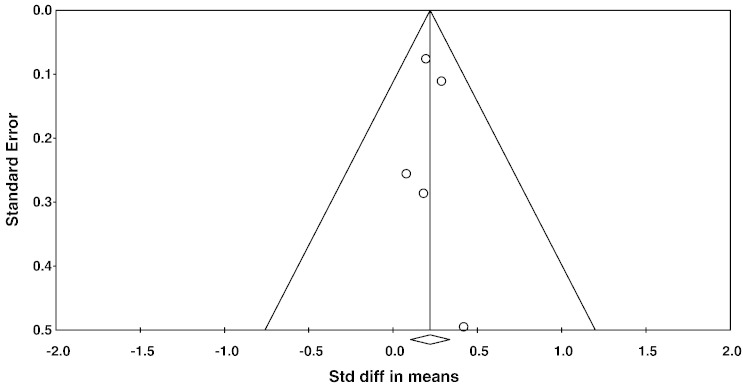
Funnel plot of standard errors plotted against effect sizes for studies in Fig. 2, in order to identify publication bias.

A meta-analysis of all five studies showed an overall small mean effect size of 0.22 (95% confidence interval (CI) = 0.10 to 0.34, p < 0.001) (Fig. 1). Studies were not significantly heterogeneous (Q_4_ = 0.956, p = 0.916, I^2^ = 0). The possibility of publication bias was assessed by calculating Begg and Mazumdar rank correlation and by inspecting a funnel plot (Fig. 2). Both suggest no publication bias: the Begg and Mazumdar rank correlation was not significant (τ = 0.100, two-tailed p = 0.81), and the funnel plot was symmetric (Fig. 2). Attempted modelling with potential confound variables (age, % female, interval between MRI and PFA assessments) was not significant.

### Local white matter volume

A total of seven studies employed a voxel-wise approach to examine WM volume locally, as summarised in Table 2.

Of six studies that examined local WM cross-sectionally, three studies did not report any significant results (Colcombe et al., 2003, Gordon et al., 2008, Honea et al., 2009). The largest study of WM volume to date, though, found that higher levels of PA were associated with increased WM volume within the corona radiata and parietal–occipital lobe (Ho et al., 2011), with each increase in PA (categorised in quintiles) associated with ~ 2–2.5% greater average WM volume. In addition, Erickson et al. (2007) reported that higher levels of PF were associated with increased frontal and corpus callosum WM volume, and Tseng et al. (2013b) found higher WM volume in temporal, parietal and occipital regions in a sample of Masters athletes compared with a sedentary control group.

One study to date has examined local WM volume before and after an exercise intervention, with the aerobic exercise group displaying a significant increase in frontal WM and corpus callosum volume compared with the stretching group (Colcombe et al., 2006).

### Global white matter lesions

A total of fourteen studies have examined the relationship between global measures of WM lesions and PFA, with results summarised in Table 3.

All studies employed a cross-sectional MRI design and primary analyses were not significant for ten studies (Burzynska et al., 2014, Carmelli et al., 1999, Fleischman et al., 2015, Frederiksen et al., 2015, Ho et al., 2011, Rosano et al., 2010, Tian et al., 2014b, Tseng et al., 2013a, Willey et al., 2011, Zheng et al., 2012). However, Saczynski et al. (2008) found that participants in the upper quartile of WML load were more likely to be physically inactive than those in the lower three quartiles; Sen et al. (2012) reported that increased PF was associated with lower total WM lesion volume, and Wirth et al. (2014) found that higher PA was associated with reduced WM lesion volume. Furthermore, Gow et al. (2012) found that a higher level of PA at baseline was associated with decreased WM lesion volume and combined rating of periventricular and deep WM lesions at 3-year follow-up.

**Fig. 3:**
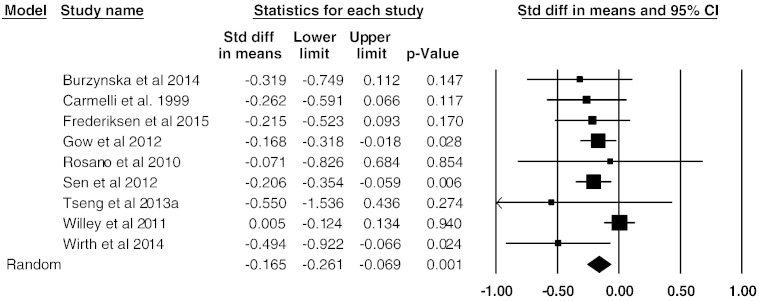
Effect sizes (Cohen's d) for total WM lesion volume: higher PFA associated with reduced WM lesion volume.

**Fig. 4:**
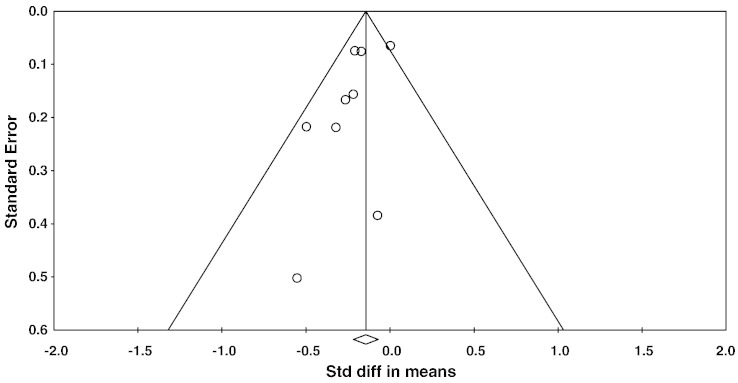
Funnel plot of standard errors plotted against effect sizes for studies in Fig. 4, in order to identify publication bias.

A meta-analysis of nine studies that assessed global WM lesion volume continuously showed an overall small mean effect size of − 0.165 (95% confidence interval (CI) = − 0.26 to − 0.07, p = 0.001 (Fig. 3). Studies were not significantly heterogeneous (Q_8_ = 10.6, p = 0.22, I^2^ = 25). Begg and Mazumdar rank correlation was not significant with τ = − 0.25 (two-tailed p = 0.35), and a funnel plot also did not indicate significant publication bias (Fig. 4). Attempted modelling with potential confound variables was not significant.

### Local white matter lesions

Two studies have examined the relationship between local measures of WM lesions and PFA, with results summarised in Table 4.

In a cross-sectional study that did not detect a difference in global WM lesion volume between Masters athletes and a sedentary control group, Tseng et al. (2013a) reported an 83% reduction in deep WMH volume.

In contrast, in a longitudinal MRI study, Podewils et al. (2007) found that a higher level of PA at baseline was associated with an increased rate of periventricular and deep WM lesion progression.

### Global white matter microstructure

Only one study examined the relationship between PFA and global measures of WM microstructure using DTI, with results summarised in Table 5.

Gow et al. (2012) reported that higher levels of PA were associated with increased FA at 3-year follow-up, with no significant relationships for diffusivity values.

### Local white matter microstructure

A total of eight studies have examined the relationship between PFA and WM microstructure locally using DTI, with results summarised in Table 6.

Seven studies examined WM microstructure cross-sectionally. Of seven studies that examined FA, three studies reported that higher levels of PFA were associated with increased FA: within the corpus callosum (Johnson et al., 2012), superior longitudinal fasciculus and arcuate fasciculus (Liu et al., 2012), and superior longitudinal fasciculus, superior corona radiate, inferior fronto-occipital fasciculus and inferior longitudinal fasciculus (Tseng et al., 2013a). Of three studies that examined MD, findings were not significant for two studies (Johnson et al., 2012, Marks et al., 2011), while Tseng et al. (2013a) reported decreases in MD in non-overlapping regions to their FA results, within the cingulum and posterior thalamic radiation. Only one study examined AD and RD, with Johnson et al. (2012) finding that reductions in RD accompanied increases in FA.

In an interventional study, Voss et al. (2012) did not find any group-level difference for FA, AD or RD. However, greater percentage change in fitness was associated with significant increases in prefrontal, parietal and temporal FA within the exercise group, with no significant relationships detected within the control group. Furthermore, correlations were significantly different between groups for prefrontal and temporal FA.

## Discussion

### Summary

Over recent years, MRI has become an increasingly popular research technique in the PFA field, and a growing number of studies are now using MRI to investigate the relationship between PFA and WM measures in healthy older adults. Overall, we view the evidence to date with cautious optimism; while many studies have found that higher levels of PFA are associated with greater WM volumes, reduced volume and severity of WM lesions or improved measures of WM microstructure, a number of negative findings have also been published. Meta-analyses of global measures of WM volume and WM lesion volume further support this standpoint, demonstrating significant, but small, effect sizes.

### Anatomy of findings

A key outstanding question identified by this review is the extent to which the relationship between PFA and WM structure is anatomically specific. First, it has been hypothesised that PFA is associated with global measures of WM structure. Despite several negative reports, our meta-analyses found that higher levels of PFA were significantly associated with higher global WM volume and smaller global volume of WM lesions, although effect sizes were small in both cases. Some support for a global effect can also be drawn from the positive relationship observed using DTI between PA levels and global FA (Gow et al., 2012), although more studies of this nature would be necessary for a more confident conclusion.

Second, it is hypothesised that the relationship between PFA and WM structure is localised to specific regions. A recent review of MRI studies that examined the relationship between PFA and grey matter volume reported consistent evidence within the hippocampus and frontal cortex (Erickson et al., 2014). While our review did not identify consistent evidence to support localised WM results within the temporal lobe, it does highlight some complementary results with regard to the frontal lobe. For example, higher PF levels have been associated with greater volumes in prefrontal WM tracts cross-sectionally (Erickson et al., 2007), a 6-month aerobic exercise intervention has been shown to lead to increases in volumes in anterior WM compared with a stretching program (Colcombe et al., 2006), and improvements in PF have been reported to correlate with increases in prefrontal FA following a one-year aerobic exercise intervention (Voss et al., 2012). However, while it is encouraging that positive findings have been reported across different WM measures and study designs, there has not yet been consistent replication of such findings.

While few studies to date have examined WM measures both globally and locally, it is important to note that global and local relationships between PFA and WM are not mutually exclusive. In fact, in a review of age-related changes in cognition, Bennett and Madden (2014) found that there was evidence to support both global and tract-specific changes in WM microstructure. In the case of PFA and WM structure, the evidence, although more sparse, seems to indicate a similar combination of global and localised effects. To further develop this discussion, it would be desirable for future studies to report both global and local changes associated with PFA.

### Methodological considerations

Studies included in this review varied in their focus, quality, design and participant demographics — limiting the extent to which results are directly comparable.

A formal quality assessment revealed that, overall, studies were of good quality (Supplementary Material: Table S1). All studies provided a good description of sample characteristics and PFA–WM findings, however, there was variation in the extent to which the methods used to assess PFA and WM outcomes were detailed, reporting of PFA and WM outcome measures for the included participants, sample size (range 15–1787), and reporting of exact p values. In addition, there was variation in the extent to which characteristics of excluded participants were described. Often, limitations reflect the relationship between PFA and WM not being the primary focus of a study, rather than the overall quality of the paper. For the two intervention studies (Colcombe et al., 2006, Voss et al., 2012), risk of selection bias was judged to be unclear, as the methods for random sequence generation and allocation concealment were not specified. Performance bias and detection bias were judged to be low-risk: blinding of participants and trainers is typically not feasible in aerobic exercise interventions, while automated imaging analysis techniques minimise the possibility researcher bias. In addition, in Colcombe et al. (2006) WM analyses were performed by a researcher who was blind to the group assignment of each individual. Attrition bias was also judged to be low-risk/unclear.

With regard to design, although twenty-one of twenty-four studies administered a MRI scan at a single time-point, the order of and interval between MRI and PFA assessments varied from PA assessment occurring 3.2 years prior to MRI scan (Gow et al., 2012) to PA assessment being administered 8–11 years after MRI scan (Tian et al., 2014a). For both global WM volume and global WM lesion volume, though, meta-regressions between effect size and the interval between time-points did not reveal significant associations. Only two studies examined WM measures before and after an aerobic exercise intervention (Colcombe et al., 2006, Voss et al., 2012), of 6-months and 12-months in length, respectively. While these studies reported some encouraging findings, further studies are needed to offer insights into the optimal frequency, intensity, time and type of exercise for WM health.

With regard to participant characteristics, studies included in this review were notably heterogeneous in terms of mean age (range 65 to 83 years) and gender distribution (range 0–100% female), and also varied in terms of the inclusion and exclusion criteria employed.

Advancing age is associated with reductions in WM volume, increasing severity of WM lesion and an accelerated decline in WM microstructure (Bennett and Madden, 2014, Gunning-Dixon et al., 2009); and a key outstanding question concerns the trajectory of the association between WM structure and PFA with age. One possibility is that greater levels of PFA are particularly beneficial in later life. Indeed, in a study of adults aged between 55 and 79 years that did not detect a main effect of PF, it was reported that age-related WM volume loss in frontal, parietal and temporal cortices was reduced as a result of PF (Colcombe et al., 2003). Such findings concur with a meta-analysis of studies examining cognitive function in older adults, which found that adults aged between 55 and 65 years benefitted least from PA interventions, compared with both adults aged between 66 and 70 years and 71 and 80 years (Colcombe and Kramer, 2003). However, in our analyses of global WM volume and global WM lesion volume, meta-regressions with age did not reveal significant associations between effect size and mean age. Furthermore, given it is possible that the brain's ability to adapt and change could actually wane with age, further research into the effect of age on the relationship between PFA and WM structure is warranted.

While beneficial effects of PFA on cognitive function have been reported in both male-only (Schuit et al., 2001, Van Gelder et al., 2004) and female-only (Weuve et al., 2004, Yaffe et al., 2001) populations, a meta-analysis of intervention studies in older adults found that cognitive benefits were significantly greater in studies in which the majority of participants were female (Colcombe and Kramer, 2003). Whether the relationship between PFA and WM structure is different in males and females, though, is not yet clear. For example, in contrast to cognitive findings, in a study of 715 participants, of whom 54% were female, the protective effect of PF on WM lesions was found to be only significant in males (Sen et al., 2012). Also, for both global WM volume and global WM lesion volume, the meta-regression with percentage of female participants in this review was not significant, and more research is needed.

Studies also varied in terms of inclusion and exclusion criteria. For example, some observational and intervention studies limited their sample to participants who displayed low levels of PA at baseline, while studies that administered fitness tests often employed strict criteria regarding vascular risk factors. It is important to note that results from such studies may not necessarily generalise to wider populations. In contrast, cohort studies that administered questionnaire-based measures of PA typically employed less stringent criteria, and such differences in inclusion and exclusion criteria may lead to a systematic bias in findings.

Finally, while the links between PFA and cognition are well documented (Bherer et al., 2013, Colcombe and Kramer, 2003, Smith et al., 2010), it is important to highlight that few studies to date have directly explored whether such links are mediated by the effects of PFA on WM structure. Encouragingly, in a path model, Wirth et al. (2014) demonstrated that current PA had a beneficial effect on global cognitive functioning via the mediation of WM lesions. However, exercise-induced increases in FA were not associated with improvements in backward digit span performance in an interventional study (Voss et al., 2012). Further studies examining domain-specific effects will be of great interest to the field.

### Mechanisms of action

A number of pathways may explain the observed relationships between PFA and WM structure. First, PFA may lead directly to improved WM structure. This hypothesis is supported by interventional studies that have shown that a 6-month aerobic exercise intervention leads to increases in volumes compared with a stretching program (Colcombe et al., 2006), and that improvements in PF over the course of a 12-month program correlate with increases in FA (Voss et al., 2012). Such a direct link may be mediated by a number of possible neurobiological mechanisms.

For example, cerebrovascular health and perfusion play an important role in WM health. In addition to periods of hypoxia, damage to the vascular wall, leakage of fluid into the surrounding tissue and disruption of the blood–brain-barrier have all been identified as contributing factors to the formation of WM lesions (Pantoni, 2002, Schmidt et al., 2007). PFA, however, has well-known beneficial effects on the vascular system, including the preservation of arterial elasticity and wall integrity, reduction in arterial stiffness and blood pressure (McDonnell et al., 2013). PFA may thus be protective against potentially damaging cerebrovascular decline. Indeed, MR angiography studies have shown an association between the number and integrity of small blood vessels and aerobic fitness in older adults (Bullitt et al., 2009), as well as many studies reporting increased capillary density with an exercise in animals (Black et al., 1990, Ding et al., 2006, Isaacs et al., 1992, Swain et al., 2003). As such, improved vascular health and cerebral perfusion as a result of PFA may contribute positively to WM health and structure via improved oxygen and nutrient delivery.

The brain's response to exercise involves several neurotrophic factors, including brain-derived neurotrophic factor (BDNF), insulin-like growth factor 1 (IGF-1) and vascular endothelial growth factor (VEGF). The strongest evidence implicates BDNF, which is increased in the expression in the rat brain in response to exercise (Neeper et al., 1996), as well as being positively linked with exercise-induced change in hippocampal volume in humans (Erickson et al., 2012) and improved functional connectivity (Voss et al., 2010). BDNF is especially interesting due to its role in the growth and survival of many neuronal subtypes, as well as in synaptic plasticity and normal axonal pruning (Cao et al., 2007, Singh et al., 2008). In addition to providing neuroprotection against hypoxic–ischaemic insult in an age-dependent fashion (Cheng et al., 1997), BDNF promotes the regeneration of injured axons in the adult rat brain (Mamounas et al., 2000) and has been shown to have a neuroprotective influence on white matter in both rodents and humans (Husson et al., 2005, Weinstock-Guttman et al., 2007). Higher secretion of BDNF in multiple sclerosis patients has been linked with higher WM volume, as well as higher inflammatory activity (Weinstock-Guttman et al., 2007), suggesting protective up-regulation. Increased expression of BDNF in response to PFA may thus have a neuroprotective influence, positively promoting WM structure and integrity. It is notable, though, that many genes that are up-regulated in response to exercise interact with BDNF. IGF-1 is a potent survival factor for neurons and oligodendrocytes, and has also been linked to improved functional connectivity after exercise, along with BDNF and VEGF (Voss et al., 2013). While the evidence is strongest for the effects of BDNF in the exercise response, it is likely that its effects are supported by both IGF-1 and VEGF and it will be important for future studies to include measures of all these factors.

Though not consistently linked, there is also some evidence to suggest an association between exercise and proliferation of oligodendrocyte progenitor cells (OPCs), which continue to generate new oligodendrocytes throughout adulthood, allowing for continued myelination. For example, in a study of voluntary exercise, running increased the number of immature and mature oligodendrocytes in the spinal cord of the mouse (Krityakiarana et al., 2010), and OPC proliferation has been reported to increase in the hippocampus by 30% after 7 days of running (Matsumoto et al., 2011). As such, the extent of axonal myelination may alter in response to exercise, mediated via increased oligodendrocyte proliferation and differentiation.

PFA may also lead to improved WM structure via third variables. For example, PFA is associated with general improvements in health and wellbeing, including positive effects on the cardiovascular system, body composition and mental health, with many of these factors having also been shown to be related to WM structure in ageing populations (Raz and Rodrigue, 2006). Several of the cross-sectional MRI studies included in this review included possible confounding variables as additional covariates in their analyses, in order to examine the degree to which such factors influence the relationship between PFA and WM structure. While, in the majority of analyses, results remained significant after taking such factors into account (Benedict et al., 2013, Gow et al., 2012, Sen et al., 2012, Wirth et al., 2014), notably Ho et al. (2011) reported that PA was no longer statistically correlated with WM volume after BMI was included in their model. In both studies that examined the effects of an aerobic exercise intervention, a control group that participated in stretching and toning classes meant that effects could not be attributed to increased social engagement associated with an exercise program (Colcombe et al., 2006, Voss et al., 2012). Moving forward, it will be important for future studies to systematically examine a range of factors hypothesised to be protective or detrimental to WM structure in the ageing process to examine the relative weight and inter-dependency of factors.

Finally, compromised WM structure may have an impact on an individual's ability to participate in physical activity, and indeed, an increased risk of impaired mobility, gait and balance dysfunction has been identified in individuals with WM changes (Srikanth et al., 2009, Starr, 2003, Wakefield et al., 2010). WM integrity may itself be a propagator of reduced physical activity, and the causal directionality of the two factors may be reversed.

## Conclusions

The study of the relationship between PFA and WM structure is still in its infancy. From the studies reviewed here, a promising tendency towards a positive relationship between PFA levels and WM structure emerged. Although encouraging, we remain cautious in our conclusions due to the small size of effects and a number of negative findings. Throughout this review, we have called attention to areas warranting further research. Specifically, the development of this research area would benefit from studies: (1) that further examine the anatomical specificity of the relationship between PFA and WM; (2) that offer insights into the optimal frequency, intensity, time and type of exercise; (3) that address how gender and age mediate the PFA–WM relationship; (4) that explore whether PFA-related effects on WM translate to cognition; and (5) that distinguish between biological mechanisms which may lay behind such effects (e.g. vascular health, cerebral perfusion, and neurotrophic factors). Ultimately, although many questions remain unanswered, PFA remains a promising candidate in the search for factors that can reduce or delay the deteriorating effects of age on WM structure.

## Figures and Tables

**Table 1:** Studies of global white matter volume.

Study	N	Mean age	% female	Design	PFA assessment	MRI assessment	Significant results
*Cross-sectional MRI*							
Benedict et al. (2013)	331	75	49.5	T1: PA, MRI	Q — no. of activities/week; divided into four groups	WM (% ICV)	↑ WM a
Bugg and Head (2011)	52	69.0 ± 6.7	71.2	T1: MRI, T2: PA; T1–T2: 2.2 y	Q — average MET hrs/week over 10 y; divided into two groups	WM	No significant results
Burns et al. (2008)	64	72.7 ± 6.3	53.1	T1: PF, MRI	FT — VO_2_ peak	WM (% ICV)	No significant results
Gow et al. (2012)	673	70	47.3	T1: PA; T2: MRI; T1–T2: 3.2 y	Q — 6-point scale	NAWM (% ICV)	↑ WM b
Tseng et al. (2013b)	20	73.4 ± 4.8	25.0	T1: PA, MRI	Q — divided into masters athletes and sedentary	WM (% ICV)	No significant results c

Abbreviations — FT: fitness test; ICV: intracranial volume; NAWM: normal appearing white matter; Q: questionnaire; T1: time-point 1; T2: time-point 2; T1–T2: interval between time-point 1 and time-point 2; WM: white matter; y: years; ↑ indicates a significant positive relationship between PFA and MRI measures.

a After co-varying for sex. Results remained significant following additional co-varying for education, serum LDL concentration, fasting plasma glucose, mean arterial blood pressure and abdominal visceral fat volume.

b % female based on complete sample of 691 participants. After co-varying for age and sex. Results did not remain significant following additional co-varying for IQ, social class and disease.

c In the same sample, raw global WM was not significantly different between masters athletes and sedentary participants.

**Table 2:** Studies of local white matter volume.

Study	N	Mean age	% female	Design	PFA assessment	MRI assessment	Significant results
*Cross-sectional MRI*							
Colcombe et al. (2003)	59	66.5 ± 5.3	55.0	T1: PF, MRI	FT — VO_2_ peak	VBM	No significant results
Erickson et al. (2007)	54	69.6 (58–80)	100	T1: PF, MRI	FT — VO_2_ peak	VBM	↑ frontal, ↑ corpus callosum
Gordon et al. (2008)	40	71.5 ± 4.7	57.5	T1: PF, MRI	FT — VO_2_ max	VBM	No significant results
Ho et al. (2011)	226	77.9 ± 3.6	57.5	T1: PA, MRI	Q — kcal/week over 2 weeks; divided into quintiles	TBM	↑ corona radiata, ↑ parietal–occipital a
Honea et al. (2009)	56	73.3 ± 6.2	58.9	T1: PF, MRI	FT — VO_2_ peak	VBM	No significant results b
Tseng et al. (2013b)	20	73.4 ± 4.8	25.0	T1: PA, MRI	Q — divided into masters athletes and sedentary	VBM	↑ temporal, ↑ parietal, ↑ occipital

*Longitudinal MRI*							
Colcombe et al. (2006)	59	66.5 (60–79)	55.0	T1: PF, MRI; T2: PF, MRI; T1–T2: 6 m	I–E: 60 min aerobic 3 × week C: 60 min stretching 3 × week	VBM	↑ frontal, ↑ corpus callosum

Abbreviations — C: control; E: exercise; FT: fitness test; I: intervention; Q: questionnaire; m: months; T1: time-point 1; T2: time-point 2; T1–T2: interval between time-point 1 and time-point 2; TBS: tensor-based morphometry; VBM: voxel-based morphometry; ↑ indicates a significant positive relationship between PFA and MRI measures.

a After co-varying for age, sex and education. Results were no longer significant following additional co-varying for BMI.

b VBM was also performed within a limbic ROI, with no significant results.

**Table 3:** Studies of global white matter lesions.

Author	N	Mean age	% female	Design	PFA assessment	MRI outcome	Significant results
*Cross-sectional MRI*							
Burzynska et al. (2014)	88	65 ± 4	62.5	T1: PF, MRI	FT — VO_2_ max	VOL	No significant results a
Carmelli et al. (1999)	148	72.7 ± 2.1	0	T1: PA, MRI	Q	VOL	No significant results
Fleischman et al. (2015)	167	80.1 ± 6.7	79.0	T1: PA, MRI	A — total daily activity	VOL	No significant results
Frederiksen et al. (2015)	282	73.1 ± 5.1	58.2	T1: MRI; T2: PA	Q — divided into active and inactive	RAT	No significant results
Gow et al. (2012)	676, 685	70	47.3	T1: PA; T2: MRI; T1–T2: 3.2 y	Q — 6-point scale	VOL, RAT	↓ VOL, ↓ RAT b
Ho et al. (2011)	226	77.9 ± 3.6	57.5	T1: PA, MRI	Q — kcal/week over 2 weeks; divided into quintiles	RAT	No significant results
Rosano et al. (2010)	30	81.0 ± 3.6	73.3	T1: PA; T2: PA; T3: PA, MRI; T1–T2: 1 y; T2–T3: 2 y	I (T1–T2) and Q (T3) E: T1–T2 > 150 min walking/week, T2–T3 continued activity; C: T1–T2 education, T2–T3 < 20 min activity/week	VOL	No significant results
Saczynski et al. (2008)	1787	75.7 ± 5.5	58.2	T1: PA, MRI	Q — h MVPA/week; divided into inactive and active	RAT (upper quartile)	↓ RAT
Sen et al. (2012)	715	65 ± 8	54.0	T1: PF, MRI	FT — VO_2_ estimate	VOL	↓ VOL c
Tian et al. (2014a)	276	72.9 ± 2.7	58.7	T1: MRI; T2: PA; T1–T2: ~ 8–11 y	Q — kcal/week; divided into three groups	VOL (median split)	No significant results
Tseng et al. (2013a)	20	73.4 ± 4.8	25.0	T1: PA, MRI	Q — divided into masters athletes and sedentary	VOL	No significant results
Willey et al. (2011)	1238	70 ± 9	59.6	T1: PA; T2: MRI; T1–T2: 6 y	Q — MET score over 2 weeks; divided into quartiles	VOL	No significant results
Wirth et al. (2014)	92	75.2 ± 5.6	63.0	T1: PA, MRI	Q — kcal/12 months	VOL	↓ VOL d
Zheng et al. (2012)	287	77.8 ± 4.5	53.7	T1: PA, MRI	Q — h/week	VOL (median split)	No significant results

Abbreviations — C: control; E: exercise; FT: fitness test; I: intervention; Q: questionnaire; RAT: rating; T1: time-point 1; T2: time-point 2; T1–T2: interval between time-point 1 and time-point 2; VOL: volume; y: years; ↓ indicates a significant negative relationship between PFA and MRI measures; ↑ indicates a significant positive relationship between PFA and MRI measures.

a WM lesion volume was significantly associated with an accelerometry-derived measure of moderate-to-vigorous PA.

b % female based on complete sample of 691 participants. After co-varying for age and sex. Total volume remained significant, but total rating did not remain significant, following co-varying for age, sex, IQ, social class and disease.

c After co-varying for age and sex. Results remained significant after additional adjustment for hypertension, diabetes, smoking, cholesterol, BMI, beta-blocker and calcium-channel-blocker treatment.

d After co-varying for age, sex and education. Results remained significant after additional adjustment for subjective memory functioning, depression or socioeconomic status.

**Table 4:** Studies of local white matter lesions.

Author	N	Mean age	% female	Design	PFA assessment	MRI outcome	Significant results
*Cross-sectional MRI*							
Tseng et al. (2013a)	20	73.4 ± 4.8	25.0	T1: PA, MRI	Q — divided into masters athletes and sedentary	VOL-P, VOL-D	↓ VOL-D

*Longitudinal MRI*							
Podewils et al. (2007)	60	77.3 ± 5.8	61.7	T1: PA, MRI; T2: MRI; T1–T2: 5 y	Q — kcal/week over 2 weeks	RAT-P, RAT-D	↑ RAT-P, ↑ RAT-D a

Abbreviations — C: control; E: exercise; FT: fitness test; I: intervention; Q: questionnaire; RAT-D: rating — deep white matter; RAT-P: rating — periventricular white matter; T1: time-point 1; T2: time-point 2; T1–T2: interval between time-point 1 and time-point 2; VOL-D: volume — deep white matter; VOL-P: volume — periventricular white matter; y: years; ↓ indicates a significant negative relationship between PFA and MRI measures; ↑ indicates a significant positive relationship between PFA and MRI measures.

a Model adjusted for age, sex, ethnicity, education, APOE genotype and corresponding baseline WM lesion score. Additional adjustment for other cardiovascular risk factors and follow-up time did not affect the results.

**Table 5:** Studies of global white matter microstructure.

Study	N	Mean age	% female	Design	PFA assessment	MRI assessment	Measure	Significant results
*Cross-sectional MRI*								
Gow et al. (2012)	691	70	47.3	T1: PA; T2: MRI; T1–T2: 3.2 y	Q — 6-point scale	Tractography (global — 12 tracts)	FA	↑ global a
MD	No significant results b
AD	No significant results
RD	No significant results

Abbreviations — AD: axial diffusivity; FA: fractional anisotropy; MD: mean diffusivity; Q: questionnaire; RD: radial diffusivity; T1: time-point 1; T2: time-point 2; T1–T2: interval between time-point 1 and time-point 2; y: years; ↑ indicates a significant positive relationship between PFA and MRI measures.

a After co-varying for age and sex. Results were no longer significant following additional co-varying for IQ, social class and disease.

b After co-varying for age and sex. Results did not change following additional co-varying for IQ, social class and disease.

**Table 6:** Studies of local white matter microstructure.

Study	N	Mean age	% female	Design	PFA assessment	MRI assessment	Measure	Significant results
*Cross-sectional MRI*								
Burzynska et al. (2014)	88	65 ± 4	62.5	T1: PF, MRI	FT — VO_2_ max	ROI on TBSS skeleton (temporal, CC, CING, SLF)	FA	No significant results a
Johnson et al. (2012)	26	64.8 ± 2.8	53.8	T1: PF, MRI	FT — composite (VO_2_ peak, FT time, 1-min HR recovery)	TBSS	FA	↑ CC
TBSS (FA significant)	MD	No significant results
TBSS (FA significant)	AD	No significant results
TBSS (FA significant)	RD	↓ CC
Liu et al. (2012)	15	66.2 ± 5.8	46.7	T1: PA, MRI	Q — min/week aerobic over 10 y; divided into two groups	Voxel-wise	FA	↑ SLF, AF^b^
Marks et al. (2011)	15	66.2 ± 5.8	46.7	T1: PF, MRI	FT — VO_2_ peak	ROI (cingulum)	MD	No significant results
Tian et al. (2014a)	276	72.9 ± 2.7	58.7	T1: MRI; T2: PA; T1–T2: ~ 8–11 y	Q — kcal/week; divided into three groups	ROI (SLF, UNF)	FA	No significant results c
Tian et al. (2014b)	164	82.9 ± 2.6	57.1	T1: PF, MRI	FT — 400 m walk time	ROI (CING)	FA	No significant results
Tseng et al. (2013a)	20	73.4 ± 4.8	25.0	T1: PA, MRI	Q — divided into masters athletes and sedentary	TBSS	FA	↑ SLF, SCR, IFOF, ILFs
						MD	↓ CING, PTR

*Longitudinal MRI*								
Voss et al. (2012)	70	64.9 ± 4.5	64.3	T1: PF, MRI; T2: PF, MRI; T1–T2: 1 y	I–E: 40 min walking 3 × week C: 60 min flexibility 3 × week	ROI on TBSS skeleton (frontal, temporal, parietal, occipital)	FA	No significant results
AD	No significant results
RD	No significant results

Abbreviations — AD: axial diffusivity; AF: arcuate fasciculus; C: control; CC: corpus callosum; CING: cingulum; E: exercise; FA: fractional anisotropy; FT: fitness test; I: intervention; IFOF: inferior fronto-occipital fasciculus; ILF: inferior longitudinal fasciculus; MD: mean diffusivity; PTR: posterior thalamic radiation; Q: questionnaire; RD: radial diffusivity; ROI: region-of-interest; SCR: superior corona radiata; SLF: superior longitudinal fasciculus; T1: time-point 1; T2: time-point 2; T1–T2: interval between time-point 1 and time-point 2; TBSS: tract-based spatial statistics; UNF: uncinate fasciculus; y: years; ↓ indicates a significant negative relationship between PFA and MRI measures. ↑ indicates a significant positive relationship between PFA and MRI measures.

a Exploratory whole-brain TBSS also showed no significant results. FA within a temporal ROI was associated with an accelerometry-defined measure of light PA.

b No significant results were detected with voxel-wise TBSS analysis. In a ROI analysis of the cingulum in the same sample, a positive relationship between VO_2_ peak and FA was reported (Marks et al., 2011).

c In a sub-sample of 164 participants, 400 m walk time at T1 was not associated with FA within superior longitudinal fasciculus and uncinate fasciculus (Tian et al., 2014b).

## Appendix A Supplementary data

Supplementary material Figure S1: Search criteria. Figure S2: Identification and attrition of papers. Table S1: Assessment of the focus and quality of included studies.
