# Supplementary material for: A systematic review of MRI studies examining the relationship between physical fitness and activity and the white matter of the ageing brain
Source: Neuroimage. 2016 May 1;131:81–90. doi: 10.1016/j.neuroimage.2015.09.071 (PMC4851455; doi:10.1016/j.neuroimage.2015.09.071)
Supplement: Supplementary file 1 — Supplementary material Figure S1: Search criteria. Figure S2: Identification and attrition of papers. Table S1: Assessment of the focus and quality of included studies. [file mmc1.docx]

**Supplementary Material**

**Figure S1: Search criteria.**

The following search strategy was used for MEDLINE and modified for other databases.

1. exp Exercise/
2. exp Physical Fitness/
3. “Physical Activity”.mp
4. 1 or 2 or 3
5. exp Magnetic Resonance Imaging/
6. exp Brain/
7. 4 and 5 and 6
8. limit 7 to “middle aged (45 plus years)”
9. limit 8 to (journal article or letter)

**Figure S2: Identification and attrition of papers.**

**Table S1. Assessment of the focus and quality of included studies**

| Study | Focus | PFA Outcome | WM Outcome | Sample | Reporting |
| --- | --- | --- | --- | --- | --- |
| (Benedict et al., 2013) | Moderate | Good | Moderate ^1^ | Good | Good |
| (Bugg and Head, 2011) | Moderate | Good | Moderate ^2^ | Good | Good |
| (Burns et al., 2008) | Good | Good | Good | Good | Moderate ^4^ |
| (Burzynska et al., 2010) | Good | Good | Good | Good | Good |
| (Carmelli et al., 1999) | Good | Moderate ^1^ | Good | Good | Moderate ^4^ |
| (Colcombe et al., 2003) | Good | Good | Good | Good | Good |
| (Colcombe et al., 2006) | Good | Good | Good | Good | Good |
| (Erickson et al., 2007) | Moderate | Moderate ^1^ | Good | Good | Moderate ^4^ |
| (Fleischman et al., 2015) | Moderate | Good | Good | Good | Good |
| (Frederiksen et al., 2015) | Moderate | Moderate ^1^ | Good | Good | Good |
| (Gordon et al., 2008) | Good | Good | Good | Good | Good |
| (Gow et al., 2012) | Good | Good | Good | Good | Good |
| (Ho et al., 2011) | Good | Good | Good | Good | Good |
| (Honea et al., 2009) | Good | Good | Good | Good | Good |
| (Johnson et al., 2012) | Good | Good | Good | Moderate ^3^ | Good |
| (Liu et al., 2012) | Good | Good | Good | Moderate ^3^ | Moderate ^4^ |
| (Marks et al., 2011) | Good | Good | Good | Moderate ^3^ | Moderate ^4^ |
| (Podewils et al., 2007) | Good | Good | Good | Good | Good |
| (Rosano et al., 2010) | Moderate | Good | Moderate ^1^ | Good | Good |
| (Saczynski et al., 2008) | Moderate | Moderate ^1^ | Moderate ^2^ | Good | Moderate ^4^ |
| (Sen et al., 2012) | Good | Good | Good | Good | Good |
| (Tian et al., 2014a) | Moderate to Good | Good | Moderate ^1,2^ | Good | Good |
| (Tian et al., 2014b) | Good | Good | Good | Good | Good |
| (Tseng et al., 2013a) | Good | Good | Good | Partial ^3^ | Moderate ^4^ |
| (Tseng et al., 2013b) | Good | Good | Good | Good | Good |
| (Voss et al., 2012) | Good | Good | Good | Good | Good |
| (Willey et al., 2011) | Good | Good | Moderate ^2^ | Good | Good |
| (Wirth et al., 2014) | Good | Good | Good | Good | Moderate ^4^ |
| (Zheng et al., 2012) | Moderate | Good | Good | Good | Good |

The **focus** of the study considered whether both PFA and WM were primary outcome measurements, and if a hypothesis or aim *directly* relating PFA to WM was clearly described.

**PFA and WM Outcomes** considered the description, validity and reporting of the outcome measure. **Sample** took into account sample size and description of sample characteristics. **Reporting** covered the description of PFA-WM findings and reporting of actual p values (not required for non-significiant voxelwise studies). Good quality indicates that the majority of criteria considered were judged to be fulfilled. It is important to note that quality was judged with regard to the examination of the relationship between PFA and WM – this may not reflect the overall quality of the paper, as often the primary focus of the study was not the relationship between PFA and WM.

^1^ limited description of assessment method; ^2^ limited reporting of outcome measure; ^3^ limited sample size (n < 30); ^4^ exact p values not reported.
